# Sutureless Amniotic Membrane Transplantation in a Pediatric Patient with Acute Toxic Epidermal Necrolysis

**DOI:** 10.4274/tjo.galenos.2019.13333

**Published:** 2019-12-31

**Authors:** Zeynep Baş, Ömür Uçakhan Gündüz

**Affiliations:** 1 Ankara University, Faculty of Medicine, Department of Ophthalmology, Ankara, Turkey

**Keywords:** Amniotic membrane transplantation, dry eye, ocular surface, toxic epidermal necrolysis

## Abstract

The purpose of this case report is to describe a new surgical method for sutureless placement of the amniotic membrane with a symblepharon ring in a pediatric patient with acute toxic epidermal necrolysis (TEN). A 1-year-old girl developed severe ocular surface inflammation with large corneal and conjunctival epithelial defects secondary to TEN. She was treated by applying a large (4 cm x 4 cm) amniotic membrane graft and non-sterile symblepharon ring under sedoanalgesia at bedside in the intensive care unit. The ocular surface was completely epithelized by post-treatment week 6 in the right and week 8 in the left eye. Two years after amniotic membrane transplantation, both eyes were quiet with no symblepharon, scar formation, or limbal stem cell deficiency. Performing bilateral amniotic membrane transplantation under a symblepharon ring at bedside provided sufficient acute coverage of the ocular surface and led to excellent clinical outcomes by reducing inflammation and protecting the ocular surface.

## Introduction

Toxic epidermal necrolysis (TEN) and Stevens-Johnson syndrome (SJS) are severe adverse drug reactions that predominantly involve the skin and mucous membranes. They are considered to be two ends of the same spectrum, differing only by their extent of skin detachment. Both are rare diseases, with an approximate incidence of 1 to 2 per 1,000,000 annually.^1^ SJS and TEN are both immune-mediated diseases caused by cytotoxic CD8+T lymphocyte response. The histopathology of SJS/TEN lesions shows that T cells respond via interferong and Fas ligand pathway, leading to keratinocyte apoptosis. Drugs are identified as the main cause of SJS and TEN in most cases, but *Mycoplasma pneumoniae* and herpes simplex virus infections are also well documented causes. Genetic background may also have an impact on risk of developing SJS/TEN. Recently, associations between HLA genotypes and drug hypersensitivity have been demonstrated in various ethnic groups.^2^ Chung et al.^3^ described strong relationships between HLA-B1502 and carbamazepine, HLA-B5801, and allopurinol.

SJS and TEN are severe and life-threatening diseases with an estimated mortality rate of 1-5% for SJS and 25-35% for TEN.^4^ Clinical findings include a prodromal symptom of fever and malaise, followed by the development of generalized, tender cutaneous eruptions. Common ocular manifestations of TEN include conjunctivitis, as well as conjunctival and corneal epithelial defects and ulcerations secondary to conjunctival inflammation. Goblet cells, lacrimal ducts, and meibomian glands may be damaged.^5^ If left untreated, these acute changes may ultimately lead to recurrent or persistent corneal epithelial defects and symblepharon formation. Severe dry eye and limbal stem cell deficiency may subsequently develop in the chronic stage.^6^ The ongoing inflammatory process on the ocular surface tends to be relentless and prolonged even after the patients are discharged from the hospital.^7^ Although intervention in the acute stage has more favorable ocular outcomes, treatments also exist for the chronic sequelae of SJS and TEN. Patients with SJS/TEN are poor candidates for traditional penetrating keratoplasty due to the development of cicatrizing lid disorders and severe ocular surface diseases. For these patients, limbal allografting and Boston keratoprosthesis may provide some visual recovery despite limbal stem cell deficiency and corneal conjunctivalization.^8^

Early evaluation and treatment of patients with SJS and TEN are critical. Recent literature data show that amniotic membrane transplantation (AMT) can suppress inflammation and facilitate healing if done in the acute phase of TEN.^7^^,9,10^ The amnion has immunomodulatory effects and promotes epithelialization. The amnion’s anti-inflammatory mechanism of action may be due to downregulation of inflammatory cytokines released by activated lymphocytes and promotion of leukocyte apoptosis.^11^

Various AMT techniques have been described previously, as traditional AMT is a time-consuming and a laborious surgery that is difficult to perform on patients who are unstable for surgical interventions because of systemic complications. It should also be taken into account that extensive eyelid sloughing in these patients makes the surgical area unfit for traditional AMT, and multiple surgeries are needed. Recently, a sutureless amniotic membrane fixation method was described, which utilized different materials to secure the membrane in the fornices. Ma et al.^12^ described a technique for sutureless application of amniotic membrane in SJS patients using sterile intravenous tubing. Kara.^13^ utilized a feeding tube to make a modified ocular surface ring in a chemical ocular surface burn patient.

In this case report, we describe a method for sutureless placement of amniotic membrane on the bulbar and palpebral conjunctiva that has been previously used for acute ocular burns but is novel in the setting of ocular TEN.^14^ To the best of our knowledge this is the first report documenting the use of this novel technique in a TEN patient.

## Case Report

**Figure 1:**
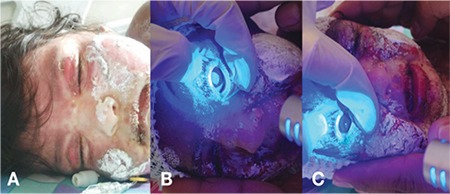
Ocular manifestations of acute toxic epidermal necrolysis: A) Sloughing and erythema on eyelid skin; B) Fluorescein-stained corneal epithelial defect; C) Eyelid margin sloughing and conjunctival injection

A 1-year-old girl was admitted to the Ankara University Pediatric Emergency Department with the suspicion of TEN. Her history revealed that she had received a measles, mumps, and rubella vaccine 13 days before admission. The day after vaccination, she developed seizures and was transferred to another pediatric emergency department. Under suspicion of febrile convulsion, she was treated with phenobarbital, levetiracetam, and cefuroxime. Seizure did not recur, and on day 9 she was discharged from the hospital. Three days after initiation of treatment, the patient developed a raised, maculopapular rash on her body, together with mucosal involvement. She was admitted to the Ankara University Pediatric Infection Unit with suspected Stevens-Johnson Syndrome. Upon admission, the anticonvulsant medications were discontinued and she was started on intravenous (IV) prednisolone 2 mg/kg/day and IV immunoglobulin 2 g/kg/day. On her second day in hospital, she was evaluated by the ophthalmology unit. On examination at bedside, the patient was observed to have severe bilateral bulbar and palpebral conjunctival inflammation, desquamation, and epithelial defects. The corneal epithelial defects measured 1x1 mm in the right eye and 7x8 mm in the left eye (Figure 1). The patient was started on aggressive lubrication with preservative-free artificial tears, as well as cyclosporine ophthalmic emulsion 0.05% (Restasis^â^, Allergan, Ireland) and loteprednol ophthalmic suspension 0.5% (Lotemax^â^, Bausch&Lomb, USA) 4 times a day to both eyes.

**Figure 2:**
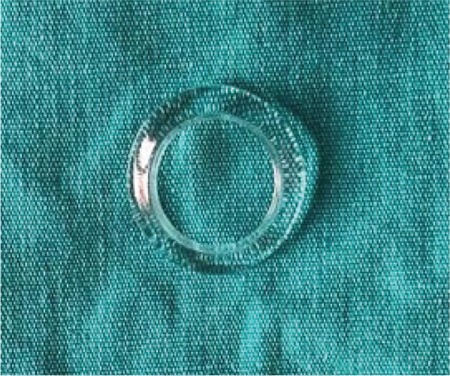
Small non-sterile symblepharon ring, size 18 mm, polymethyl methacrylate (IMKA, Ankara, Turkey)

The patient remained in critical condition, which prevented her from leaving the pediatric unit for surgery. During this period, it was noted that her systemic condition was worsening despite systemic treatment, so she was treated with infliximab (Remicadeâ, Essex GmbH, Germany) 5 mg/kg as single-shot therapy. The patient’s severe clinical condition, intense laryngeal desquamation, and edema precluded her from receiving general anesthesia. On post-admission day 3, it was decided to perform AMT at bedside under sedoanalgesia. The placenta was retrieved intact and processed under sterile conditions. The chorioamnion was stripped from the placenta, and following antibiotic decontamination, the amniotic membrane was separated from the chorion, cut into 4-cm squares and mounted on nitrocellulose backing paper as previously described.^15^ After instilling 1 drop of proparacaine (Alcaine^®^, Alcon, USA), the amniotic membrane was spread onto the ocular surface epithelial side up. Since there was severe epidermal desquamation, we had difficulty even holding the eyelids open and instead of securing the amniotic membrane with sutures, a symblepharon ring was gently placed on the ocular surface in the superior and inferior fornix. The symblepharon ring (open fornix conformer) was chosen according to the height of palpebral apertures and was big enough to safely sit in the fornices. It was made of polymethyl methacrylate and had a diameter of 18 mm (IMKA, Ankara, Turkey) (Figure 2). The rest of the membrane was tucked into the palpebral conjunctiva using forceps and the excess was trimmed with Westcott scissors. The same procedure was performed on the contralateral eye. Postoperatively, moxifloxacin 0.5% (Vigamox^®^, Alcon, USA) 4 times a day and tacrolimus ointment 0.03% (Protopic^®^, Astellas, USA) 2 times a day were added to topical treatment.

**Figure 3:**
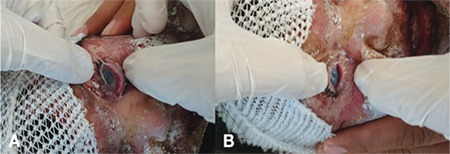
Postoperative day 3: Amniotic membranes wrapped around a symblepharon ring in the A) Right eye and B) Left eye

**Figure 4:**
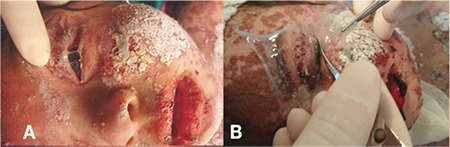
Postoperative day 7: A) Amniotic membrane has begun to erode on the left side; B) Amniotic membrane transplantation was repeated at bedside with the same procedure on both eyes

**Figure 5:**
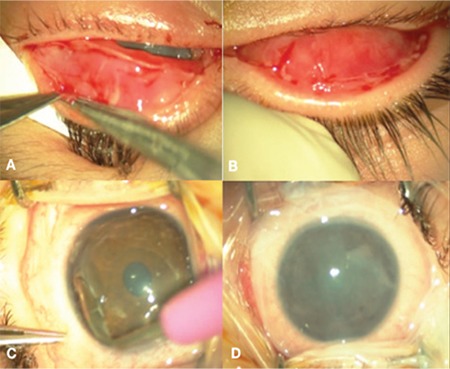
Postoperative 1 month, examination under general anesthesia: Epithelization has begun on the A) left and B) right palpebral conjunctiva. In the left eye, C) peripheral keratectomy was performed and D) amniotic membrane transplantation was repeated for the third time, this time under general anesthesia

**Figure 6:**
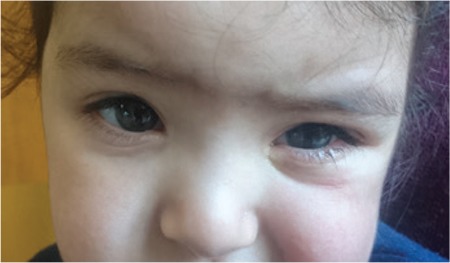
At 6-month follow up, the patient had grade 1 corneal haze in her left eye

**Figure 7:**
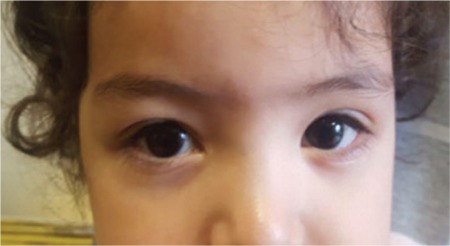
At 2-year follow-up, the patient exhibited no corneal haze or other sequelae

On postoperative day 3, the amniotic membranes and symblepharon rings were still in place (Figure 3). Disintegration of the amniotic membrane was noticed and the procedure was repeated on both eyes on day 7 (Figure 4). The patient was seen 1 month later. Her systemic condition was stable, so she was examined under general anesthesia. Examination of her right eye on day 37 showed the corneal defect was healed and the tarsal conjunctiva was epithelized, and a bandage contact lens (Pure Vision, Bausch & Lomb, USA) was applied. In the left eye, the symblepharon ring and the old disintegrated membrane were removed, and a 7x7 mm persistent corneal epithelial defect was observed. Peripheral keratectomy was performed and a new amniotic membrane was sutured onto the cornea using 10-0 nylon sutures, and a bandage contact lens was placed (Figure 5). The corneal defect in the left eye was healed on day 53 and remained that way through the remainder of the follow-up. The patient was kept on cyclosporine, tacrolimus, artificial tears and ointments for 3 months, followed by tapering of all medications. At postoperative 6 months, there was no ectropion and the conjunctival fornices were preserved without residual inflammation in both eyes. There was mild corneal haze in left eye (Figure 6). At 2-year follow-up, both corneas were clear with no residual scars (Figure 7). No other scarring sequelae occurred.

## Discussion

Toxic epidermal necrolysis is a severe immunologic dermatobullous condition with high morbidity and mortality. It is characterized by widespread erythema, necrosis, and bullous detachment of the epidermis and mucous membranes. It exists on the same spectrum as SJS, but is characterized by more than 30% body surface area detachment.^16^ It is believed that drugs or metabolites, acting as receptors, bind to surface of keratinocytes and cause them to become antigenic and stimulate cytotoxic CD8+T cell response.^17^ In our case, the immune reaction was considered to be triggered by the use of phenobarbital. SJS and TEN have long been associated with the administration of barbiturates, with the greatest risk occurring within the first two months of treatment.^4^

The incidence of TEN is very low but the ocular sequelae can be blinding. Rapid recognition and prompt withdrawal of the offending drug is essential. The main clinical presentations are conjunctival inflammation, keratinization of the ocular surface, and symblepharon formation. Recent literature data indicate that early intervention with AMT in the hyperacute phase gives better long-term results in terms of ocular surface and forniceal stability.^7^^,10^ Amniotic membrane suppresses inflammation, prevents ulcer formation, and promotes healing of the ocular surface.^11^ A delay in the treatment may result in corneal stem cell deficiency and sight-threatening cicatricial complications.^18^ However, securing the amniotic membrane onto the ocular surface with sutures at bedside in intensive care units is technically difficult and poses several challenges.^7^ Anchoring the amniotic membrane requires complex and time-consuming suturing of multiple amnion pieces to the eyelids, fornix, and limbus that requires bolsters and stitch removal from the patient’s eyelids and ocular surface. In order to address some of these problems, sutureless techniques in SJS patients have been published recently by Shay et al.^7^, Pruet et al.^9^, Cheung et al.,^10^ and Ma et al.^12^ Cheung et al.^10^ used specially made symblepharon rings that are not commercially available and hard to find, possibly causing operations to be delayed. Shay et al.^7^ utilized ProKera rings, but the diameter of the ProKera only covers the cornea and perilimbal conjunctiva, thus making the fornices vulnerable to symblepharon formation.^19^ Ma et al.^12^ employed an intravenous tube, but the circular shape of the tube may not be an adequate fit for the oval contour of the fornices.

Our technique minimized symblepharon formation because of increased coverage of the amnion membrane thanks to the broad shape of the symblepharon rings. Symblepharon rings are effective in keeping the fornices intact in conjunctival cicatricial diseases. The ring prevents adhesions and forniceal contractures without touching the cornea. Liang et al.^14^ used a similar sutureless technique on burn patients and reported higher reepithelialization rates, shorter operation time, and lower symblepharon rates in the sutureless AMT group. To our knowledge, this technique was never performed in patients with SJS/TEN.

Patients with acute TEN often do not receive AMT during the hyperacute phase because of the lack of standardized protocol, high mortality risk associated with general anesthesia, and difficulty in performing this extensive surgery. Our technique can be performed at the bedside without the need for general anesthesia or operating room conditions. This minimizes the delay in AMT and is less invasive for the patient. In addition, amniotic membrane coverage of the entire conjunctival surface is crucial to maximizing benefit; therefore, patients undergoing traditional AMT only to the bulbar conjunctiva may still develop chronic sequelae of SJS and TEN. A possible disadvantage is that the amniotic membrane and ring may come loose from the fornices. However, the simplicity of the method allows easy manipulation of the membrane.

In the absence of banked cryopreserved amnion and due to the pressing nature of the patient’s condition, we decided to use fresh amniotic membrane. There may be a number of disadvantages to this, the most important being the theoretical risk of disease transmission. Another difficulty is the need to find a suitable donor fast enough in advance of surgery to allow processing and testing and coordination with the obstetrics and gynecology department.^15^ No such problems arose in our case.

The use of a symblepharon ring with amniotic membrane to cover the ocular surface and fornices without the use of sutures or tissue glue as described herein is fast, nontraumatic, technically easy, and seems to yield final outcomes comparable to those achieved with conventional AMT methods. The results of this study are in agreement with recently published reports that AMT performed in the acute phase of TEN is vital to prevent sight-threatening cicatrizing sequelae associated with ocular manifestations of the disease.
